# Female trainees believe that having children will negatively impact their careers: results of a quantitative survey of trainees at an academic medical center

**DOI:** 10.1186/s12909-018-1373-1

**Published:** 2018-11-13

**Authors:** Cindy Kin, Rachel Yang, Pooja Desai, Claudia Mueller, Sabine Girod

**Affiliations:** 1 Department of Surgery, Stanford University School of Medicine, Stanford, CA 94305 USA; 2 Department of Medicine, University of California Los Angeles Medical Center, Los Angeles, CA 90095 USA

**Keywords:** Graduate medical education, Gender, Work-family conflicts, Parenthood

## Abstract

**Background:**

Medical training occurs during peak childbearing years. However, the intense workload, long work hours, and limited financial compensation are potential barriers to having children during this time. Here, we aimed to identify gender-based differences in beliefs and experiences of having children during graduate medical education. We hypothesized that both genders face significant challenges, but women are more likely to experience stressors related to work-family conflicts.

**Methods:**

We administered an anonymous web-based survey to all trainees at an academic medical center. Primary outcomes were gender differences in beliefs and experiences of having children during training. Multivariate logistic regression was performed using independent variables of gender, specialty type (surgical vs. medical), and parental status.

**Results:**

In total, 56% of trainees responded (60% women, 40% men; *n* = 435). Women were more often concerned about the negative impact of having children and taking maternity leave on their professional reputation and career. The majority of women expressed concern about the potential negative impact of the physical demands of their jobs on pregnancy. Among parents, women were more likely than men to be the primary caregivers on weeknights and require weekday childcare from a non-parent.

**Conclusions:**

Women face greater work-related conflicts in their beliefs and experiences of having a family during graduate medical education. Trainees should be aware of these potential challenges when making life and career decisions. We recommend that institutions employ solutions to accommodate the needs and wellbeing of trainees with families while optimizing training and workload equity for all trainees.

**Electronic supplementary material:**

The online version of this article (10.1186/s12909-018-1373-1) contains supplementary material, which is available to authorized users.

## Background

Despite the challenges of graduate medical education, including intense work, long hours, high stress levels, and limited financial compensation, an increasing proportion of trainees are choosing to have children. [1, 2] Current trainees face a different set of challenges than trainees of prior generations. First, as a greater proportion of medical trainees are women, those who wish to become mothers during training face the challenge of balancing their professional obligations with the medical needs of pregnancy and childbirth. Second, professionals in the millennial generation are far more likely to be dual-career couples, which poses much greater logistical challenges for having children. [3] Thus, in contrast to male trainees with stay-at-home spouses that were more common decades ago, today’s male trainees are more likely to have working spouses and thus face the challenge of balancing their professional obligations with increased responsibilities of parenthood.

The impact of childbearing on trainees’ personal and professional lives as well as on training programs is significant. The rules and culture of many specialties that affect having children during training have not adapted to the changing demographics and realities of current trainees. As a result, female physicians are more likely to delay parenthood relative to other professional women, potentially because of the risk of obstetrical complications due to the physical stress of training, limited parental leave and childcare options, and workweek requirements from certifying boards. [4–7] It is unclear to what extent these factors affect male trainees in their family planning decisions. Understanding the challenges and concerns of both male and female trainees is critical to the ability of training programs and graduate medical education in the U.S. to successfully foster the next generation of physicians.

The aims of this study were to identify differences between men and women in their concerns and experiences regarding having children during training. We hypothesized that despite both genders facing significant challenges associated with having children during training, women would (1) more likely believe that parenthood would negatively affect their careers, (2) more often postpone parenthood due to job-related factors, and (3) bear a greater burden of primary childcare responsibility than their male counterparts.

## Methods

The investigators designed a series of survey questions to probe the experiences of trainees about having children (a blank copy of this can be seen in Additional file 1). The Stanford University Institutional Review Board approved the study. Ten volunteers tested the survey for clarity and the investigators adjusted questions based on their feedback. All resident and fellow trainees at Stanford University Medical Center received an invitation to complete the survey via a link in their email to a secure and HIPAA-compliant online platform (QuestionPro, San Francisco, CA); those who completed it received a $10 gift certificate. We sent an email reminder after one week, and closed the survey after two weeks.

Our primary outcome measures were gender differences in concerns and experiences of having children during graduate medical education. Specifically, we analyzed the results from questions 8b-8 k, 10–13, 16b-16 k, 17–20, 28–30, 35, and 36 (Supplement). The relevant survey questions are detailed in Tables 1, 3, and 4. The outcome variable was the proportion of respondents who responded “strongly agree” or “agree” to the statements detailed in Tables 1 and 4, where indicated. We used Chi-square tests to analyze categorical variables, considering *p* < 0.05 significant. We performed multivariate logistic regression for outcome variables that differed significantly between male and female respondents, using independent variables of gender, specialty type (medical vs. surgical), and parental status. The outcome variables for the regression analyses are detailed in Tables 2 and 5. We used STATA/SE 13.1 statistical software.

**Table 1:** Concerns about Having Children among Male and Female Trainees

	Overall %	Male %	Female %	*p*-value
Adequacy of resources for having children (% responding “strongly agree” or “agree”)^a^				
I don’t have time for a/nother child	84.2	80.1	87.1	NS
I don’t have money for a/nother child	72.9	76.5	70.7	NS
I don’t have enough stability in my life for a/nother child	63.8	65.1	63.1	NS
I don’t have the adequate resources for childcare	75.9	83.0	71.5	0.03
I am worried about physical demands of my job and pregnancy (females only)	–	–	77.9	–
Impact of children on professional life (% responding “strongly agree” or “agree”)^a^				
I am worried I will be a burden on colleagues by taking parental leave	55.4	42.5	63.3	< 0.0001
I do not feel that I can take parental leave	52.1	58.4	48.2	NS
I am worried about how I will be perceived professionally if I have a/nother child	28.6	10	41.2	< 0.0001
I am worried about the impact of (more) children on my future career	40.2	26.8	49.5	< 0.0001
Level of interest in having children				
I think about having children sometimes, often, or all the time	81.8	74.4	86.7	0.005
I discuss the topic of having children with friends and colleagues (response: yes)	85.5	74.1	92.7	< 0.0001
How do you feel when you think about having (more) children? (% responding “strongly agree” or “agree”)^a^				
Happy and excited	73.6	77.9	70.9	NS
Anxious	78.4	73.8	81.1	NS
It’s a source of stress in our relationship	20.4	21.8	19.6	NS
Sad	14.0	12.2	15.2	NS
Emotionally neutral	11.7	11.0	12.3	NS
How worried are you about the possibility of never having children? (non-parents only)				
Very or somewhat worried	64.3	46.6	75.5	< 0.0001
Not worried, although do want kids	35.7	53.4	24.5	< 0.0001
Don’t know if want kids	7.0	8.7	6.0	NS
Don’t want kids	1.2	0	2	NS

^a^Response choices were on a Likert scale: strongly agree, agree, neither agree nor disagree, disagree, strongly disagree, prefer not to say

**Table 2:** Multivariate Models of Resident Perceptions of Having Children During Residency, Using Independent Predictors of Gender, Specialty Type (Surgical vs. Medical), and Parental Status

	Female		Surgical		Parent	
	OR (95% CI)	*p*	OR (95% CI)	*p*	OR (95% CI)	*p*
I am worried that having children will negatively affect how I will be perceived professionally	7.5 (4.02, 13.8)	0	2.24 (1.28, 3.95)	0.005	2.05 (1.20, 3.52)	0.009
I am worried that I will be a burden on colleagues by taking parental leave	2.32 (1.51, 3.59)	0	2.29 (1.34, 3.89)	0.002	0.57 (0.36, 0.92)	0.022
I am worried about the negative impact of (more) children on my future career	2.76 (1.76, 4.33)	0	1.10 (0.67, 1.83)	NS	1.20 (0.75, 1.94)	NS
I do not have the adequate resources for childcare	0.5 (0.30, 0.85)	0.01	1.18 (0.63, 2.2)	NS	0.49 (0.29, 0.82)	0.007
I do not feel like I can take parental leave	0.66 (0.42, 1.02)	NS	2.9 (1.69, 4.98)	0	0.85 (0.53, 1.37)	NS
Parental leave is not available to me	0.33 (0.18, 0.59)	0	2.60 (1.39, 4.87)	0.003	1.00 (0.53, 1.88)	NS

## Results

In total, 435 of the 776 (56%) residents and fellows who received the survey responded (60% women, 40% men). The age distribution and relationship status were similar between genders. A greater proportion of male versus female trainees were parents (26% vs. 22%, *p* = 0.02).

The majority of men and women shared concerns about lacking the time, money, or stability to have children (Table 1). In addition, most trainees of either gender felt happy and excited (74%) but also anxious (78%) about the prospect of having children. Among trainees who were not already parents, a higher proportion of women felt sad when thinking about having children (16.8% vs. 8.5%, *p* = 0.047) and worried that they may never have children (75.5% vs. 46.6%, *p* < 0.0001) (Table 1).

The genders differed in their beliefs of how parenthood might affect their professional careers. Compared to men, a higher proportion of women worried that having a child during training would lead to negative professional perceptions of them (41% vs. 10%, *p* < 0.0001), adversely impact their future careers (50% vs. 27%, *p* < 0.0001), and burden their colleagues (63% vs. 43%, *p* < 0.0001) (Table 1). In a multivariate regression controlling for gender, specialty type (medical vs. surgical), and parental status, female gender remained predictive for the belief that having a child during training would negatively affect their professional reputations [odds ratio (OR) 7.5, 95% confidence interval (CI) 4–14, *p* < 0.0001], adversely affect their career (OR 2.8, 95% CI 1.8–4, *p* < 0.0001), and burden their colleagues (OR 2.3, 95% CI 1.5–3.6, *p* < 0.0001) (Table 3). Male gender and not having children were factors associated with the belief that they lack adequate resources for childcare. Over half of all trainees did not feel they could take family leave, although most knew it was available to them (Table 2).

**Table 3:** Training Program-related Factors Affecting Trainees’ Decisions to have Children

	Overall %	Male %	Female %	*p*-value
Perceived training program support of trainees having children				
Extremely or somewhat supportive	78	72.3	81.5	NS
Extremely or somewhat unsupportive	9.8	14.8	6.6	0.023
Neither supportive nor unsupportive	12.3	12.9	11.9	NS
Parental leave				
Parental leave is not available to me (strongly agree or agree)*	17.9	28.9	11.6	< 0.0001
How long is maternity leave?				
< 6 weeks	5.3	4.1	6.3	0.003
6 weeks	25.8	13.4	34.3	< 0.0001
> 6 weeks	17.7	11.6	21.5	0.009
Don’t know	51.2	70.9	37.9	< 0.0001
How long is paternity leave?				
2 weeks or less	35.6	37.8	34.0	NS
> 2 weeks	4.2	5.2	3.5	NS
Don’t know	60.2	57.0	62.5	NS
Do you feel this amount of leave is adequate?				
Yes	11.6	8.1	14.1	NS
No	42.1	35.5	46.9	0.02
Don’t know	44.7	55.7	37.9	0.0006
Work-week requirements for advancement				
Does your program or certifying board mandate a minimum number of workweeks per year?				
Yes	42.1	48.3	38.3	0.04
No	7.9	5.8	9.4	NS
Don’t know	50	45.9	52.3	NS
Do these rules influence your decision to have children?				
Yes	43.3	33.7	51.5	0.016
No	31.1	43.4	20.6	0.001
Don’t know	16.1	10.8	20.6	NS
When is the optimal time to have a child?				
Medical school	10.6	9.2	11.6	NS
Junior residency years	2.8	3.4	2.3	NS
Senior residency years	16.1	14.4	17.4	NS
Research residency years	28.7	29.3	28.6	NS
Fellowship	19.8	18.4	20.8	NS
In practice, first 5 years	32.2	36.2	29.7	NS
In practice, after 5 years	9.7	9.2	10.0	NS
No optimal time	48.5	40.8	53.3	0.013

*Response choices were on a Likert scale: strongly agree, agree, neither agree nor disagree, disagree, strongly disagree, prefer not to say

While 78% of all trainees reported that their training programs were at least somewhat supportive of them having children, a higher proportion of men than women felt their programs were unsupportive (15% vs. 7%, *p* = 0.02), did not believe that parental leave was available to them (29% vs. 12%, *p* < 0.0001), were unsure of the amount of leave they could take (57% of men vs. 38% of women, *p* < 0.0001), or felt the amount was inadequate (56% of men vs. 38% of women, *p* = 0.0006) (Table 3). Rules on minimum workweeks required for advancement were more likely to influence women than men in their decisions to have children (52% vs. 34%, *p* = 0.016); women were more likely to believe that there was no optimal time to have children (53% vs. 41%, *p* = 0.013).

Male and female trainees who were already parents had comparable levels of dissatisfaction with their abilities to provide adequate time (85% dissatisfied), finances (68% dissatisfied), and emotional resources (48% dissatisfied) (Table 4). Childcare responsibilities differed significantly between genders. On weekdays, the primary caregivers for male trainees’ children were more likely to be their partners (52% vs. 9%, *p* < 0.0001), whereas children of female trainees were ten-fold more likely (Table 5) to be under the care of daycare, nanny, or extended family (91% vs. 48%, *p* < 0.0001). Female versus male trainees were more often the primary caregiver on weeknights (47% vs. 17%, *p* = 0.001; OR 4.9, *p* = 0.001) and weekends (OR 2.7, *p* = 0.02) (Tables 4 and 5).

**Table 4:** Experience of Being Parents during Graduate Medical Education

	Overall %	Male %	Female %	*p*-value
Satisfaction with ability to provide adequate resources to care for child(ren) (% who “strongly agree” or “agree” with the statements)				
I am satisfied with my ability to provide adequate time to care for my child(ren)	14.9	23.8	8.8	NS
I am satisfied with my ability to provide financial resources to take care of my child(ren)	32	36.4	29.1	NS
I am satisfied with my ability to provide adequate emotional resources to take care of my child(ren)	51.6	51.2	52.9	NS
Primary Caregiver for Child(ren) on:				
Weekdays				
Trainee’s partner	27.9	52.2	8.9	< 0.0001
Other (daycare, nanny, extended family)	72.1	47.8	91.1	< 0.0001
Weeknights				
Trainee	33.3	17.4	47.4	0.003
Trainee’s partner	61.9	76.1	49.1	0.01
Other (daycare, nanny, extended family)	4.8	6.5	3.5	NS
Weekends				
Trainee	53.3	41.3	63.2	NS
Trainee’s partner	41.9	52.2	33.3	NS
Other (daycare, nanny, extended family)	4.8	6.5	3.5	NS

**Table 5:** Multivariate Models of Childcare Responsibilities among Residents who are Parents, using Independent Factors of Gender and Specialty Type (Medical vs. Surgical)

	Female			Surgical		
	OR	95% CI	p	OR	95% CI	p
Primary caregiver on weekdays						
Trainee’s partner	0.10	0.03, 0.29	< 0.0001	0.67	0.20, 2.28	NS
Other	10.28	3.48, 30.32	< 0.0001	1.50	0.44, 5.10	NS
Primary caregiver on weeknights						
Trainee	4.87	1.89, 12.54	0.001	0.22	0.06, 0.84	0.027
Trainee’s partner	0.27	0.11, 0.64	0.003	3.83	1.14, 12.82	0.03
Other	0.55	0.09, 3.41	NS	0.94	0.10, 8.92	NS
Primary caregiver on weekends						
Trainee	2.67	1.16, 6.19	0.022	0.17	0.05, 0.52	0.002
Trainee’s partner	0.42	0.18, 0.97	0.04	5.66	1.94, 16.53	0.002
Other	0.55	0.09, 3.41	NS	0.94	0.10, 8.92	NS

## Discussion

This study examined gender differences in the concerns of trainees of having children during graduate medical education. The most striking differences between male and female trainees were in their beliefs about the impact of parenthood on their professional careers. With adjustment for specialty type, female trainees were more likely to worry that having children would engender poor professional perceptions of them and negatively impact their future careers. These concerns are valid interpretations of not only the cultural milieu of academic medicine, but also the pervasive biases within our society at large, which has led to the “motherhood penalty.” [8] The unfortunate reality is that trainees who are pregnant or mothers face workplace discrimination. [9] Program directors believe parenthood negatively impacts female trainees’ more than male trainees’ work. [10] These biases are not unique to the medical field, and affect working women across industries and socioeconomic classes. A job applicant experiment demonstrated that compared to women without children, mothers are considered less competent and committed, require higher exam scores, are held to stricter performance and punctuality rules, are offered lower salaries, and are less likely to be promoted or hired. In contrast, fathers are considered more committed and promotable than non-fathers, face less strict punctuality standards, and are offered higher salaries. [11] Subconscious or conscious recognition of these pervasive biases in our society is likely to also affect medical trainees.

The majority of trainees were happy but anxious about the prospect of having children. This anxiety could be attributed to concerns about the ability to provide for children due to limited time, finances, and stability. Training program-related concerns also likely influence trainees’ decisions regarding children. Only 12% of trainees felt that the length of parental leave was adequate, with many male trainees unaware they were eligible for leave. In addition, trainees feared that having children would prevent them from graduating on time. These concerns are warranted, as 40% of pediatric residents had to extend their training due to maternity leave. [12–14]

The postpartum stressors examined in our study included feelings of guilt over colleagues’ increased workloads due to parental leave, which occurred more commonly in females. Other studies have shown that peers evaluate female trainees poorly after pregnancy and often feel anger and resentment toward pregnant residents due to their increased workload. [15, 16] Although men were less likely to be concerned about the professional ramifications of having children, they were more likely to feel unable to take parental leave.

In addition to concerns of negative professional ramifications that are commonly experienced by women across industries, women in graduate medical education face some unique challenges due to the nature of the work. Most female trainees worried whether the physical demands of their jobs were compatible with a healthy pregnancy, consistent with studies reporting that female trainees tend to defer pregnancy due to concerns for complications from work-related stressors. [13] These concerns are not unfounded, as increased physical stress is associated with pregnancy complications. [6, 17] In fact, female trainees are at higher risk for preterm labor, miscarriage, intrauterine growth restriction, placental abruption, and hypertension compared with spouses of male residents and the general population [16–23]; complication rates among surgeons exceed women in the lowest income brackets. [24]

Another stressor that especially affected women was the greater burden of childcare. Female versus male trainees more often were the primary caregiver on weeknights and required weekday childcare services, increasing the financial burden. [25] Other stressors include an unpredictable work schedule and the difficulty of breastfeeding after maternity leave. [26, 27] The inherent stress of early parenthood is compounded by the lack of institutional resources, as only 38% of academic hospitals have on-site childcare and less than 60% have lactation spaces. [10] Workplace stigma against pumping breast milk at work and the lack of time to do so may impact the duration of breastfeeding. [28]

A limitation of this study is the potential for response bias, as trainees who feel more strongly about these matters may be more likely to respond. Another potential confounder is the amount of financial pressure experienced by our respondents may be particularly pronounced due to the high cost of living in the Bay Area.

Our study illustrates the need for trainees, faculty, training programs, and certifying specialty boards to recognize these challenges, and proactively address them. Most trainees would like to welcome children into their lives, but have legitimate concerns over whether having children is compatible with training. These concerns affect both male and female trainees, as the increase in dual-career families leads to increased difficulty with balancing career and family. Indeed, two-thirds of millennials find it difficult to manage both the personal and professional aspects of their lives. [3] We offer the following recommendations for the academic medical establishment to address these needs of trainees in the pipeline:

1. *Training programs should have formal written parental leave policies and comprehensive education about them.* [29] The majority of programs lack formal policies that address parental leave, coverage for residents on leave, and work expectations for pregnant trainees. [29–31] Policies vary widely among programs, and are often not well-communicated. [32]
2. *Programs should allow all trainees to take at least six weeks of paid parental leave and up to six additional weeks of unpaid leave; programs should also protect pregnant trainees from physically taxing work schedules to mitigate potential risks to maternal and fetal health.* The American Academy of Pediatrics advocates that each residency program have a parental leave policy that at least conforms to the Family and Medical Leave Act, which entitles employees to 12 weeks of leave within one year of birth or placement of a foster or adopted child, with at least 6–8 weeks paid. [12, 33] Longer length of maternity leave is associated with improved maternal mental and physical health. [34–38]
3. *Faculty mentors should help set realistic expectations.* The effects of motherhood on occupational prestige and wages attenuate over time, such that working mothers in their 50s are on par with childless women. [8] The implication for academic medicine is that women’s career paths may follow a different pattern of academic productivity over time than their male colleagues.
4. *Institutions should foster a supportive work environment through coverage plans for trainees on leave, dedicated lactation spaces and time, and affordable childcare.* Paying clinical associates to cover the workload of a trainee on parental leave would alleviate the burden on other trainees. Establishing dedicated lactation spaces and awareness of the need for pumping breaks would protect the choice of mothers to continue breastfeeding. [39] Affordable on-site childcare with extended hours may be the only way many trainees can logistically have children with their on-call responsibilities.
5. *A competency-based model for advancement should be based on the Accreditation Council for Graduate Medical Education milestones, rather than the number of work weeks.* This individualized approach to education and competency allows for more flexibility in the length of training while ensuring that trainees are ready for independent practice. [40]

## Conclusions

Finding solutions to allow trainees to take longer parental leaves while maintaining quality and equity for other trainees is necessary for a sustainable graduate medical education workforce. Physicians should not be expected to defer having children until after training, risk their or their infants’ health due to heavy workloads, or forgo critical baby bonding time. In the long run, the willingness and ability of academic medical centers to accommodate these needs while maximizing education and workload equity will create a more stable and robust workforce. [41]

## Additional file

Additional file 1: Blank copy of the survey. (DOCX 88 kb)

## Abbreviations

CI: Confidence interval
OR: Odds ratio
