# Supplementary material for: Female trainees believe that having children will negatively impact their careers: results of a quantitative survey of trainees at an academic medical center
Source: BMC Med Educ. 2018 Nov 13;18:260. doi: 10.1186/s12909-018-1373-1 (PMC6234638; doi:10.1186/s12909-018-1373-1)
Supplement: Supplementary file 1 — Blank copy of the survey. (DOCX 88 kb) [file 12909_2018_1373_MOESM1_ESM.docx]

Survey

Consent: If you wish to proceed with this anonymous and confidential survey, please indicate that you agree. You will receive an Amazon.com gift card in the amount of $10 if you complete the survey. If you do not wish to proceed with the survey, please close this window.

1. Please indicate your gender.
   1. Male
   2. Female
   3. Other
   4. Prefer not to say
2. Please indicate your age group:
   1. 21-25
   2. 26-30
   3. 31-35
   4. 36-40
   5. 41-45
   6. over 46
3. Please check your current residency program. We have combined smaller programs to protect your anonymity.
   1. Anesthesia
   2. Pediatrics/Child Neurology
   3. Dermatology/Pathology/Physical Medicine & Rehabilitation
   4. Emergency Medicine
   5. Internal Medicine
   6. Neurology/Psychiatry
   7. General Surgery
   8. Obstetrics/Gynecology
   9. Surgical Specialties: Neurosurgery, Urology, Plastic Surgery, Otolaryngology, Orthopedic Surgery, Thoracic Surgery, Vascular Surgery, Ophthalmology
   10. Radiology/Radiation Oncology/Nuclear Medicine
   11. Other
4. Are you in a long-term partnership?
   1. Yes
   2. No
   3. Prefer not to say
5. Do you have children?
   1. Yes (if yes, answer questions 6-13, then skip to 20)
   2. No (if no, skip to question 14)
6. How many children do you have?
   1. 1
   2. 2
   3. 3
   4. 4
   5. 5+
   6. Prefer not to say
   7. Other
7. Are you considering having more children during your residency?
   1. Yes
   2. No
   3. I don’t know
   4. Prefer not to say
8. Regarding the decision to have more children or not, please indicate your agreement or disagreement with the following statements: (Choices: Prefer not to say, strongly disagree, disagree, neither agree nor disagree, agree, strongly agree)
   1. My partner does not want another child at this time
   2. I/we do not have enough time to take care of another child
   3. I/we do not have enough money to take care of another child
   4. I/we do not have enough stability in our lives to take care of another child
   5. I do not have adequate resources for childcare
   6. I am worried about the physical demands of my job and pregnancy
   7. I am worried about being a burden on my colleagues by taking parental leave
   8. I do not feel that I can take parental leave
   9. I am worried about how I will be perceived professionally if I have another child
   10. I am concerned about the impact of more children on my future career
   11. Family leave is unavailable to me
9. My professional obligations played a major role in the timing of having children. (Choices: Prefer not to say, strongly disagree, disagree, neither agree nor disagree, agree, strongly agree)
10. Who has primary role of taking care of your child(ren) during the following times? (Choices: me, my partner, other family members, nanny, au pair, babysitter, daycare or school)
    1. On weekdays
    2. On weeknights
    3. On weekends
11. How often do you think about having more children?
    1. I never think about it
    2. I rarely think about it
    3. I sometimes think about it
    4. I often think about it
    5. I think about it all the time
    6. Prefer not to say
    7. Other
12. When you think about the possibility of having more children, how do you feel? Please indicate whether you agree or disagree with the following statements: (Choices: Prefer not to say, strongly disagree, disagree, neither agree nor disagree, agree, strongly agree)
    1. I am happy and excited about it
    2. I am anxious about it
    3. It is a source of stress in my relationship with my partner
    4. I am sad about it
    5. I am emotionally neutral about the issue
13. Are you satisfied with your ability to provide the following resources to take care of your child(ren)? (choices: Prefer not to say, Yes, No, I don’t know, Not applicable)
    1. Time
    2. Financial resources
    3. Emotional resources
14. Why don’t you have children? (select all that apply)
    1. Prefer not to say
    2. I don’t know
    3. I prefer to not ever have children
    4. I do not have a stable long-term partner
    5. My partner does not want a child
    6. I do not feel ready to be a parent
    7. My job makes it too difficult for me to have a child
    8. I would like a child but have not been able to
    9. Other
15. Are you deferring having children because of your job?
    1. Yes
    2. No
    3. I don’t know
    4. Prefer not to say
    5. Other
16. Please indicate your agreement or disagreement with the following statements. (Choices: Prefer not to say, strongly disagree, disagree, neither agree nor disagree, agree, strongly agree)
    1. My partner does not want a child at this time
    2. I/we do not have enough time to take care of a child
    3. I/we do not have enough money to take care of a child
    4. I/we do not have enough stability in our lives to take care of a child
    5. I do not have adequate resources for childcare
    6. I am worried about the physical demands of my job and pregnancy
    7. I am worried about being a burden on my colleagues by taking parental leave
    8. I do not feel that I can take parental leave
    9. I am worried about how I will be perceived professionally if I have a child
    10. I am concerned about the impact of children on my future career
    11. Family leave is unavailable to me
17. How often do you think about having children?
    1. I never think about it
    2. I rarely think about it
    3. I sometimes think about it
    4. I often think about it
    5. I think about it all the time
    6. Prefer not to say
    7. Other
18. When you think about the possibility of having children, how do you feel? Please indicate whether you agree or disagree with the following statements: (Choices: Prefer not to say, strongly disagree, disagree, neither agree nor disagree, agree, strongly agree)
    1. I am anxious about it
    2. It is a source of stress in my relationship with my partner
    3. I am sad about it
    4. I am emotionally neutral about the issue
19. How worried are you about the possibility of never having children?
    1. I don’t want to have children
    2. I don’t know if I want to have children
    3. I do want children but I don’t worry about it
    4. I do want children and I am somewhat worried
    5. I do want children and I am very worried about it
    6. Prefer not to say
    7. Other
20. When do you think is the optimal professional time to have a child (assuming that it is possible from a personal standpoint)? Please select up to two choices.
    1. Medical school
    2. Junior resident – clinical years
    3. Senior resident – clinical years
    4. Resident – research years
    5. Fellow
    6. First 5 years of being an attending
    7. After 5 years of being an attending
    8. There is no optimal time
    9. Other (please describe)
21. Is the topic of having children something you discuss with your friends or colleagues?
    1. Yes
    2. No
    3. Prefer not to say

1. Is the topic of having children something you have discussed with your professional mentors?
   1. Yes
   2. No
   3. Prefer not to say
2. Is the topic of having children something you would like to discuss with your professional mentors?
   1. Yes
   2. No
   3. I don’t know
   4. Prefer not to say
3. Your main professional mentor is:
   1. Male
   2. Female
   3. Prefer not to say
   4. I don’t have a professional mentor
4. Do you have any role models within your field of medicine who have the type of work/life/family balance that you would like to emulate?
   1. Yes
   2. No
   3. I don’t know
   4. Prefer not to say
5. Are these role models male or female?
   1. Male
   2. Female
   3. I have both male and female role models
   4. Prefer not to say
6. Do these role models have children?
   1. Yes
   2. No
   3. I don’t know
   4. Prefer not to say
7. How would you describe your residency program’s support of residents who have children during residency?
   1. Extremely supportive
   2. Somewhat supportive
   3. Neither supportive nor unsupportive
   4. Somewhat unsupportive
   5. Very unsupportive
   6. Don’t know
   7. Prefer not to say
   8. Other
8. How much family leave does your program allow?
   1. Maternity leave
      1. 2 weeks or less
      2. 3 weeks
      3. 6 weeks
      4. 2 months
      5. 3 months
      6. more than 3 months
      7. I don’t know
   2. Paternity leave
      1. 2 weeks or less
      2. 3 weeks
      3. 6 weeks
      4. 2 months
      5. 3 months
      6. more than 3 months
      7. I don’t know
9. Do you think this is adequate time for family leave?
   1. Yes
   2. No
   3. I don’t know
   4. Prefer not to say
   5. Other
10. Does your program expect residents to take extra call to make up the number of calls one would miss during maternity or paternity leave?
    1. Yes
    2. No
    3. I don’t know
    4. Prefer not to say
11. Does your program require that residents make up the clinical time one would miss for maternity or paternity leave?
    1. Yes
    2. No
    3. I don’t know
    4. Prefer not to say
12. Other than night call, how often do you have work commitments during “off-hours”? (ie, journal club in the evenings)
    1. Never
    2. Rarely (a few times per year)
    3. Occasional (once a month)
    4. Regularly (once a week)
    5. Often (more than once a week)
    6. Don’t know
    7. Other
13. On average, how many hours do you work per week? (include both time spent in-house and on home call)
    1. <40 hours
    2. 40-49
    3. 50-59
    4. 60-69
    5. 70-79
    6. 80-89
    7. 90-99
    8. 100+
    9. Prefer not to say
14. Does your program or certifying board have a rule about the number of weeks you must work per year in order to advance?
    1. Yes
    2. No
    3. I don’t know
15. Do the rules about the number of weeks you must work per year in order to advance influence your timing of having children?
    1. Yes
    2. No
    3. I don’t know
    4. Not applicable
    5. Prefer not to say
    6. Other
16. Do you know anyone in your program who had to make up clinical time or calls due to maternity or paternity leave?
    1. Yes
    2. No
    3. I don’t know
    4. Prefer not to say
    5. Other
17. Do you know anyone in your program who was unable to graduate on time due to maternity or paternity leave?
    1. Yes
    2. No
    3. I don’t know
    4. Prefer not to say
    5. Other
18. Is there anything else you would like to add about your experiences and opinions regarding having children during residency, we encourage you to share your thoughts below.
